# Hypothalamic orexins as possible therapeutic agents in threat and spatial memory disorders

**DOI:** 10.3389/fnbeh.2023.1228056

**Published:** 2023-07-27

**Authors:** Filip Mazur, Jarosław Całka

**Affiliations:** Department of Clinical Physiology, Faculty of Veterinary Medicine, University of Warmia and Masuria in Olsztyn, Olsztyn, Poland

**Keywords:** neurophysiology, orexins, fear memory, spatial memory, navigation, CNS, memory disorders

## Abstract

Orexin-A and orexin-B, neuropeptides produced exclusively in the lateral hypothalamus, have been implicated in various functions, including memory. Their levels are elevated in certain pathological states, such as PTSD, and lowered in other states, e.g., memory deficits. Recent developments have shown the possibilities of using orexins to modulate memory. Their administration can improve the results of test animals in paradigms such as passive avoidance (PA), cued fear conditioning (CFC), and the Morris water maze (MWM), with differences between the orexin used and the route of drug administration. Blocking orexin receptors in different brain structures produces opposing effects of memory impairments in given paradigms. Therefore, influencing the orexinergic balance of the brain becomes a viable way to ameliorate memory deficits, shift PTSD-induced recall of stressful memories to an extinction path, or regulate other memory processes.

## 1. Introduction

Orexins are neuropeptides that were isolated from the central nervous system in 1998 by two separate groups of scientists. de Lecea et al. (1998) identified the mRNA encoding precursor of orexin peptides, prepro-orexin, and at the same time, Sakurai et al. (1998) identified them while searching for peptide ligands for G-protein-coupled receptors. There are two splice variants of orexins: A (OX-A), which consists of 33 amino acids with two intrachain disulphide bonds; and B (OX-B), which has a linear 28 amino-acidic structure. Their synthesis is limited to the lateral hypothalamus/perifornical area (LH/PFA) of the interbrain (Peyron et al., 1998; Date et al., 1999; Nambu et al., 1999). They bind to two orphan G-protein-coupled receptors: OX1R, which has a ten times greater affinity to Orexin-A than to Orexin-B, and OX2R, which has a similar affinity to both peptides. Their occurrence is to some extent complementary: OX1R is abundant in the locus coeruleus (LC), laterodorsal tegmental nucleus (LDT), and pedunculopontine tegmental nucleus (PPT), and OX2R is mainly expressed in the tuberomammillary nucleus (TMN), nucleus accumbens (NAc), and septal nuclei. In structures like the hippocampus or amygdala, both types of receptors are expressed generally, with one more dominant than the other (Marcus et al., 2001). Because of this vast occurrence of orexin receptors and extensive projections of orexinergic neurons throughout the neuroaxis, they regulate numerous physiological processes: feeding and energy homeostasis (Edwards et al., 1999; Volkoff et al., 1999; Yamada et al., 2000), reward system (Leinninger et al., 2011; Sakurai and Mieda, 2011; Yokobori et al., 2011), and addiction (Rao et al., 2013; Baimel et al., 2015), sleep and wake cycles (Carter et al., 2009; Tsujino and Sakurai, 2013) and, finally, learning and memory (Jaeger et al., 2002). Memory is the capacity that allows us to connect experiences and learn (Camina and Güell, 2017). It is the foundation of personality, the uniqueness of an individual, and the internal trace of our lives. Memory disorders directly affect our sense of security, as well as our comfort and that of our loved ones. It feels necessary to look for way to counteract these disorders.

Encoding is the initial phase of learning during which perceived stimuli create memory traces in the brain. A trace consists of those neurons that fire together in the first instance response to novel stimuli. Sometimes it is used interchangeably with engram, although the second term is rather defined as a more stabilized conglomerate of neurons that stores a certain episode in the long-term memory (Liu et al., 2012). After an initial trace is created, the neurons that the trace consists of may undergo various physical and biochemical changes, such as new protein synthesis, plastic events including new spikes, synapses or dendrite formation, long-term potentiation (LTP) or long-term depression (LTD), which are long-lasting increases or decreases in the signal transmission strength between two neurons, and wave oscillations between numerous structures, which form a stable engram of a particular memory. This is called the consolidation phase and the significant part of these processes occur during sleep (Teyler and DiScenna, 1986; Alvarez and Squire, 1994; McClelland, 1995; Bero et al., 2014). When a memory is consolidated and stable, it can be actively or passively recalled in a retrieval phase by changes in protein synthesis in the hippocampus (Lopez et al., 2015) and activation of some of the engram neurons, which subsequently stimulate the remaining neurons of a recalled memory engram in a way dependant on the used paradigm (Barros et al., 2000, 2001). All these phases rely heavily on interconnections between subcortical structures, such as the hippocampus, amygdala, or locus coeruleus, and cortical areas that store memories long-term, as the hippocampus mediates the sorting of positive and negative engrams and takes part in replays of cortex-based consolidated engrams of older memories during recollection (Shpokayte et al., 2022).

Owing to obvious limitations in assessing memory processes in animal models, there are two common approaches in research that evaluate either a threat or spatial memory, both of which form an episodic memory–consciously recollected memories related to personally experienced events (Ploran and Wheeler, 2017) and essential for survival in any environment. The first one forms after a certain number of repetitions of used paradigms utilizing unconditioned aversive stimuli (e.g., mild electric shock) paired with neutral contextual or auditory stimuli to condition an aversive response to the neutral stimulus (therefore creating a conditioned stimulus) in a measurable time. The latter emerges from navigating a given space with distal cues, e.g., to locate a preferred place (such as a platform in a pool, a box with a reward, or a previously unexplored maze arm).

Various medical conditions such as Alzheimer’s, Parkinson’s, dementia, post-traumatic stress disorder (PTSD), narcolepsy, anxiety, or major depressive disorder (MDD) affect, *inter alia*, these two types of memory. One of the promising courses of research in recent years refers to the role of orexins in memory processes, as many studies suggest a high level of control is exerted by the lateral hypothalamus/perifornical area over numerous memory-related structures (Flores et al., 2015). The aim of this Review is to systematize these data and to indicate directions for further studies in this field, as the manipulations of the orexin system may provide new clinical approaches in treating numerous diseases or preventing memory loss and lay the foundations for future studies concerning the mechanisms behind memory.

## 2. Fear memory

**FIGURE 1:**
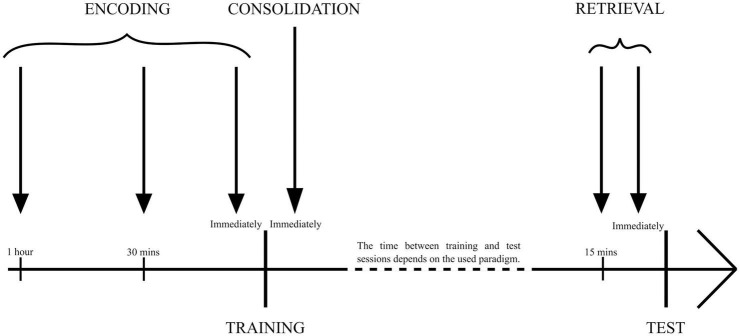
The time points of drug administration used for specific memory phase examination. Encoding: 1 h, 30 min, or immediately before training session/sessions. Consolidation: immediately after training session/sessions. Retrieval: 15 min or immediately before the test session.

Memories related to threatening stimuli and situations that cause anxiety, stress, and fear are referred to as fear memory. With numerous diseases like PTSD, generalized anxiety disorder, or obsessive-compulsive disorder, there is a continuous need to elucidate their pathogenesis and the neural physiology behind their symptoms while keeping in mind that the fearlike state that the animal models try to avert might be entirely different to what humans call fear (LeDoux, 2014). To systematize data from different studies, three time periods were differentiated, which were used for the administration of drugs to examine different memory processes. To influence an encoding phase, researchers administered drugs 1 h (Soya et al., 2017), 30 min (Telegdy and Adamik, 2002; Palotai et al., 2014), or immediately (Sears et al., 2013) before training. To study the consolidation phase, drugs were administered immediately after training (Jaeger et al., 2002; Sears et al., 2013; Flores et al., 2014; Ardeshiri et al., 2017; Mavanji et al., 2017). The retrieval phase requires drug administration 30 min (Telegdy and Adamik, 2002; Palotai et al., 2014) or immediately before test trials (Jaeger et al., 2002; Ardeshiri et al., 2017; Figure 1). Furthermore, researchers used a different approach to analyze the impact of orexin. The first was administering the peptides themselves, either to healthy animals or individuals with receptor deficiency, mimicking pathological states of various diseases. The other approach administered orexin receptor antagonists, impairing their functioning during crucial memory formation moments (Table 1).

**TABLE 1:** Research on the effect of orexins or their receptors’ antagonists on fear memory processes.

References	Animal models	Used paradigms	Substances	Administration	Conclusion
Jaeger et al., 2002	SAMP8 mice CD-1 mice	T-maze footshock avoidance Passive avoidance test	Orexin-A	After training/ before the test	OX-A administration improves memory consolidation but not retrieval.
Telegdy and Adamik, 2002	Wistar rats	Passive avoidance test	Orexin-A	30 min before training / after training / 30 min before the test	OX-A improves encoding, consolidation, and retrieval.
Sears et al., 2013	Sprague Dawley rats	Cued fear conditioning test Optogenetics	SB-334867 TCS-OX2-29 SB-334867	Before training / after training / before the test Before training Before training / after training	OX-A but not OX-B is required for fear memory encoding. Orexin neurons mediate depolarization and LA activation and as a result enhance threat learning.
Soya et al., 2013	Knockout mice: OX1R- OX2R-	Cued fear conditioning test Contextual fear conditioning test	AAV vectors	–	OX1R but not OX2R participates in fear memory formation.
Palotai et al., 2014	Wistar rats	Passive avoidance test	Orexin-B EMPA	30 min before training / after training / 30 min before the test After training / 30 min before the test	OX-B improves encoding, consolidation, and retrieval. EMPA blocked OX-B action but did not change results in the control.
Flores et al., 2015	C57BL/6J mice 129S1/SvImJ mice OX1R- knockout mice	Cued fear conditioning test Contextual fear conditioning test Novel object-recognition test Open field test Elevated plus-maze test	SB-334867 TCS-OX2-29 Orexin-A Orexin-B	After training / after each extinction session After training / after each extinction session After each extinction session After each extinction session	Weaker consolidation phase in animals with OX1R and OX2R blockade. OX1R blockade attenuated cued FC. Blockade of OX1R facilitated the extinction of threat memories and OX-A impeded it.
Flores et al., 2017	C57BL/6J mice	Contextual fear conditioning test	SB-334867	After the first extinction session	The antagonist facilitated the extinction of threat memory.
Ardeshiri et al., 2017	Wistar rats	Passive avoidance test	SB-334867 TCS-OX2-29	After training / before the test After training / before the test	Consolidation was impaired by a post-training block of OX1R but not OX2R. Blockade of each receptor before the test impaired the retrieval of threat memory.
Mavanji et al., 2017	O/A3 mice (C57BL/6J)	Two-way active avoidance test	Orexin-A	Daily after each trial	Impaired fear memory formation in orexin-deficient O/A3 mice. State improved by OX-A.
Soya et al., 2017	BAC-transgenic NAT- Cre mice Orexin-Cre mice	Optogenetics (DREADD) Cued fear conditioning test	SB-334867	1 h before the test / 30 min before the test	Blockade of OX1R resulted in a significant reduction of freezing behavior. Confirmed circuit of OXLH-LC and NA LC-LA neurons.
Dustrude et al., 2018	Sprague Dawley rats	Cued fear conditioning test DREADD	C-56 JNJ10397049 AAV vectors	–	OX1R antagonism in the CeA reduces the expression of conditioned fear.
Salehabadi et al., 2020	Wistar rats (SPS)	Contextual fear conditioning test Open field test Elevated plus maze test	SB-334867	After training/ three injections each after extinction sessions	OX1R blockade impairs threat memory formation. Antagonist normalized behavior.

### 2.1. Encoding

The first neural processes that appear in these paradigms, i.e., recognition of threats and creating fear responses, mainly involve the hippocampus, amygdala, and medial prefrontal cortex–areas that seem to be enveloped by orexinergic neurons. The lateral hypothalamus/perifornical area directly innervates hippocampal formation, with CA1, CA2, and the dentate gyrus (DG) expressing mainly OX1R, CA3 mainly expressing OX2R (Trivedi et al., 1998; Lu et al., 2000; Hervieu et al., 2001; Marcus et al., 2001; Akbari et al., 2011), and the basolateral amygdala with orexinergic fibers acting through both OX1 and OX2 receptors which, in turn, modulate central nucleus output (Bisetti et al., 2006).

In addition to its direct innervation, the LH/PFA region sends orexinergic neurons to the locus coeruleus, a nucleus involved in stress and fear responses. Activation of orexin neurons in the LC has been observed in response to aversive stimuli, such as shock, and this activation, in turn, affects the amygdala via LC-noradrenaline fibers (Chen and Sara, 2007; Sara, 2009; Soya et al., 2017).

The hippocampus plays a role in integrating contextual cues and receives input from the collaterals of sensory pathways through the ventral tegmental area (VTA). Meanwhile, the amygdala processes early aversive memory traces, and the medial prefrontal cortex facilitates memory encoding (Izquierdo et al., 2016). The wide distribution of orexins in the brain suggests that they may contribute to associative learning and, in collaboration with other neurotransmitters, participate in the selection of new memory traces for further consolidation and the formation of stable engrams.

To further support the involvement of orexin receptors in fear conditioning, experiments utilizing intracerebroventricular infusion of an OX1R antagonist called SB-334867 have shown that blocking these receptors immediately before training impairs fear response. This finding was reported by Sears et al. (2013) in the context of Pavlovian fear conditioning. The same authors showed that blocking OX2R with specific antagonist TCS-OX2-29 before training did not affect learning. In another study, an improvement in encoding was observed after OX-B ICV infusion before training, which was completely abolished by ICV infusion of the orexin receptor blocker EMPA (Palotai et al., 2014). Therefore, it might be noted that the elevated concentration of OX-B can facilitate memory encoding mainly through OX2 receptors, but it is not sufficient to sustain even a base level of learning in the case of an OX1 receptor block. On a similar basis, OX1 receptors located in the hippocampus, amygdala, and locus coeruleus are pivotal for the creation of memory traces strong enough to undergo later consolidation and long-term memory formation.

### 2.2. Consolidation

Jaeger et al. (2002) investigated the role of orexins in fear memory for the first time, using a T-maze active avoidance paradigm. They administered orexin-A intracerebroventricularly, which improved the results of 12-month-old SAMP8 mice in this paradigm in a dose-dependent manner. In 4-month-old mice, only the highest dose resulted in a better score; however, the SAMP8 strain develops memory impairments at approximately 6 months of age, which would mean that the second case showed improvement in clinically healthy individuals. Additionally, they tested the impact of OX-A ICV infusion on CD-1 mice in a passive avoidance paradigm. Both experiments showed that the peptide improves memory retention when administered immediately after training but not 24 h later. Considering that ICV administration mostly targets the hippocampus, the results are consistent with the notion that fear memory consolidation is a hippocampus-dependent process susceptible to loss over time. In another study, researchers managed to restore consolidation to base levels in mice with a genetic loss of orexins by injecting OX-A into the CA1 region, using a two-step active avoidance paradigm, further corroborating hippocampus involvement in this process (Mavanji et al., 2017). Subsequently, blocking the OX1 receptor with selective antagonist SB-334867 immediately after training impaired consolidation in contextual fear conditioning (Flores et al., 2014) and passive avoidance (Ardeshiri et al., 2017). In the first experiment, the drug was injected intraperitoneally, and in the second, directly into the basolateral amygdala. On the other hand, Sears et al. (2013) noted no effect of SB-334867 on the consolidation phase after post-training ICV infusion in cued fear conditioning. As the used doses were similar, these somewhat contradictory findings may result from different paradigms, as it was theorized that the amygdala might not be crucial for contextual conditioning as opposed to paradigms using auditory cues (Vazdarjanova and McGaugh, 1998). The involvement of orexinergic neurons in amygdala-dependent learning could benefit from further studies using c-Fos expression techniques to elucidate their engagement and amygdala response in different paradigms. The OX2 receptor seems to be significantly less involved in consolidation, as was proven by several different studies. The blockade of this receptor by ICV or intra BLA infusion of antagonist TCS-OX2-29 did not alter the performance of animal models in cued conditioning or passive avoidance (Sears et al., 2013; Ardeshiri et al., 2017), except for one study by Flores et al. (2014) in which intraperitoneal injection of the drug effectively impaired cued and contextual fear conditioning in rats. However, the dosage of TCS in this experiment was significantly higher than in the previous ones, which might have potentially blocked OX1 receptors as well, despite the high selectivity of the drug. Furthermore, the orexinergic neurons activate noradrenergic input of the locus coeruleus to the amygdala during the consolidation phase in cued and contextual fear conditioning (Soya et al., 2013). It was proven that mice lacking OX1R on noradrenergic LC fibers show impaired memory formation during cued conditioning, which can be abolished by restoring the receptors with an AAV vector. Subsequently, contextual conditioning was impaired in both OX1R and OX2R knockouts, which was not abolished by restoring the receptors. These results were further corroborated with DREADD techniques, showing that orexinergic innervation of the locus coeruleus is crucial for the activation of the amygdala in auditory-based paradigms and much less important in contextual learning (Soya et al., 2017; Dustrude et al., 2018). However, the exact circuits between the lateral hypothalamus, locus coeruleus, and amygdala in different disorder models should be investigated.

### 2.3. Retrieval

Experiments around the retrieval phase are based on the recognition of familiar stimuli, which, in turn, activates engram cells in a specific pattern. The timing of drug administration is pivotal for determining their influence on this process. Jaeger et al. (2002) administered OX-A intracerebroventricularly 24 h after training, immediately before a test session in passive avoidance and T-maze footshock avoidance paradigms, and concluded that the neuropeptide had no effect on test scores in animal models. However, OX-A infused 30 min before the test session improved the passive avoidance results in rats (Telegdy and Adamik, 2002), as was the case with OX-B in the same paradigm (Palotai et al., 2014), which may suggest that a longer period allows higher receptor saturation, which facilitates engram activation. However, more research is needed to test this hypothesis or propose a different one. On the other hand, the total blockade of either OX1 or OX2 receptor by selective antagonists administered intra BLA immediately before the test session impaired the retrieval phase in the passive avoidance paradigm (Ardeshiri et al., 2017), which suggests that both receptors take part in engram activation during fear memory recall. The study from Salehabadi et al. (2020) showed that, in Single Prolonged Stress (SPS) rats, which are the animal models of PTSD, three consecutive injections of SB-334867 into the amygdala (each after one test session, 24 h apart) could significantly decrease freezing behavior in a contextual fear conditioning paradigm. In a different study, a single injection of OX1R receptor antagonist after the first of two extinction sessions in contextual fear-conditioned mice enhanced fear extinction compared with the control group (Flores et al., 2017). Perhaps controlling orexinergic activation might prove useful in shifting memory processes toward extinction in severe anxiety cases or states such as PTSD. However, the downregulation of orexinergic innervation and its effect on memory in animal models of different disorders needs further elucidation.

## 3. Spatial memory

Navigating through a novel environment activates two processes involved in spatial memory: allocentric processing, which relies heavily on hippocampal structure and allows one to place oneself in a given space by analyzing relationships between distinct environmental cues or landmarks, and parietal cortex-dependent egocentric processing, which analyses the relationships between oneself and visible cues (O’Keefe, 1976). Different test paradigms may force animals to depend more on one of these strategies, although they still both happen simultaneously. The optimal strategy utilized by the brain involves allocentric processing of a novel environment, with egocentric processing being predominant in a well-remembered place. The two systems complement each other and are integrated by the posterior cingulate cortex (PCC) and retrosplenial cortex (RSC) (Coughlan et al., 2018). To successfully encode novel spatial information and allow memory consolidation, gamma wave activity between the hippocampus and the medial prefrontal cortex is needed during the timeframe of the working spatial memory (Spellman et al., 2015).

### 3.1. Encoding

The Morris water maze, which is predominantly used to assess spatial memory, tests the ability of animals to find and memorize the location of a transparent platform hidden in the pool in which they swim, allowing them to rest upon it. Aou et al. (2003) tested the effects of different doses of OX-A on performance in this paradigm, subsequently assessing long-term potentiation and long-term depression in Shaffer collaterals in rats. The results showed that intracerebroventricular infusion of the neuropeptide impaired spatial memory formation in all used doses. However, the drug was administered 2 h before training sessions, which deviates from other studies in this matter. The electrophysiology of hippocampal slices showed significant suppression of LTP by a higher dose, while LTD was not affected. A possible explanation for this phenomenon is that elevated OX-A increased levels of noradrenaline as well as activated GABAergic inhibitory interneurons, which, in turn, suppressed the induction of LTP. The lack of synaptic plasticity events within the medial temporal lobe resulted in the inhibited formation of memory traces, thus slowing the learning process (Sil’kis, 2013). In a different study using the same paradigm, the blockade of the OX1 receptor through the injection of SB-334867 directly into CA1 15 min before the test session significantly impaired memory encoding (Akbari et al., 2006). This may suggest that orexin A is pivotal for base-level spatial memory formation, which is on a par with the well-described OX-A-mechanism-activating ERK1/2 pathway (Ramanjaneya et al., 2009; Liu et al., 2015; Greene et al., 2020), which, in turn, triggers or strengthens LTP in the hippocampus (Peng et al., 2010). It might be useful to further elucidate the effects of orexins on spatial memory encoding at different times of drug administration to establish more concise frames for encoding studies.

### 3.2. Consolidation

The blockade of orexin receptors immediately after the training session by selective antagonist infusion into CA1 resulted in reduced time spent in the target quadrant in a Morris water maze (Akbari et al., 2006). Interestingly, it seems that the effect of this blockade can be mitigated by extended training. After antagonizing OX1 or OX2 receptors in the basolateral amygdala, the animals showed impaired spatial memory formation in the short Morris water maze paradigm, which was ameliorated with longer training (Ardeshiri et al., 2019). Furthermore, in a six-trial Morris water maze, the animal models were able to memorize the location of the hidden platform, similar to the base level learning, as opposed to the two-trial paradigm, during which the learning was significantly impaired by the OX1R blockade (García-Brito et al., 2018). The researchers subsequently assessed c-Fos protein expression in the dentate gyrus, CA2, CA2, dorsal retrosplenial cortex, prelimbic cortex, and thalamic nuclei. The immunohistochemistry data revealed that c-Fos expression in these areas was significantly higher in control animals and even higher in animals subjected to antagonist infusion and a prolonged training protocol, a finding that overlaps with the mitigation of the antagonist effect. The mechanism behind this phenomenon remains to be elucidated. Another way to alleviate the antagonist injection is intracranial self-stimulation (ICSS), as shown by the same authors (García-Brito et al., 2020). The procedure was able to compensate for receptor blockade in the Morris water maze and visual discrimination task. However, as the stimulation procedure was reported to be highly pleasurable, its effects may be explained by acting on the reward system, aside from memory formation, because the ventral tegmental area, a part of the medial forebrain bundle sending dopaminergic fibers, which is stimulated during ICSS, is innervated by orexinergic neurons and participates in memory formation processes. Likewise, the blockade of the OX1 receptor in the dorsal raphe nucleus, the most prominent serotonin source in the brain, impairs the consolidation of spatial memory (Khodabande et al., 2021). On the other hand, antagonizing the OX2 receptor with TCS-OX2-29 did not affect the results in the Morris water maze, despite the expression of OX2R. In one knockout mice experiment, animals without OX receptors in the dorsal raphe nucleus exhibited impaired spatial working memory, with locomotor activity largely intact in an exploration task and a delayed non-matching-to-place T-maze task. DRN serotonergic neurons promote wakefulness and regulate the sleep cycle (Sörman et al., 2011), as well as modulate theta rhythms in the hippocampus (Adidharma et al., 2019), which means that these results may not present a clear view of the involvement of orexins in DRN-related memory processes. What is more, the dorsal raphe nucleus is also densely innervated by noradrenergic fibers, which may excite it through a similar mechanism to orexins (Aghajanian, 1985). Therefore, to elucidate the precise effects of orexins on DRN in memory processes, further studies are required utilizing rigorous conditions to assess the overall state of the animal models.

The effect of orexins on spatial memory was also investigated in SPS rats. During one study, the control group animals showed impaired spatial memory in the Morris water maze test, decreased food intake and downregulation of OX-A. Subsequently, an upregulation of OX1 and OX2 receptors in the hippocampus was discovered, possibly as a compensatory mechanism. ICV injection of OX-A alleviated these symptoms, improving memory, enhancing appetite, and partially reversing the changes in receptor density (Han et al., 2020). Such a decrease in orexin levels was also suggested to contribute to age-related (Stanley and Fadel, 2012) and Parkinson’s disease deficits (Fronczek et al., 2012). Animal Parkinson’s models showed a correlation between declarative and spatial memory impairments and depletion of orexinergic neurons in the lateral hypothalamus/perifornical area (Oliveira et al., 2020). Destroying dopaminergic and orexinergic neurons by injecting 6-OHDA intrastriatally and Saporin intra-LH, respectively, and subsequently evaluating the orexin system with immunofluorescence techniques revealed that maximal memory deficiency was induced by the joint destruction of 88% of dopaminergic and 29% of orexinergic fibers. The role of orexins alone in this model needs to be further examined.

### 3.3. Retrieval

Spatial memory retrieval is the least researched phase in the recent literature. It has been shown to be affected by a pre-test session OX1R antagonist SB-334867 injection directly into CA1 in the Morris water maze paradigm (Akbari et al., 2006). Each used dose impaired memory retrieval in animal models which manifested in less time spent in the target quadrant, while visual and motor functions showed no alterations compared with the control group. Direct intervention in the trisynaptic loop diminished the ability to utilize an allocentric strategy in navigating the environment. However, injecting either SB-334867 or TCS-OX2-29 directly into the basolateral amygdala did not have any effect on the retrieval in rats (Ardeshiri et al., 2019). The results corroborate the notion that the role of the amygdala in the retrieval phase is time-dependent (Liang et al., 1982) and continually diminishes to the point that activating this structure via the orexinergic system does not produce any effect. Furthermore, no significant difference between the control group and experimental groups was found in the Morris water maze test using either OX1R or OX2R blockade in the dorsal raphe nucleus directly before the test session (Khodabande et al., 2021). Considering the differences between the effects of orexins on various structures and the time-dependent manner of these effects, more studies are needed to draw consistent conclusions. The effects of orexins or their antagonists were briefly summarized (Table 2).

**TABLE 2:** Research on the effect of orexins or their receptors’ antagonists on spatial memory processes.

References	Animal models	Used paradigms	Substances	Administration	Conclusion
Aou et al., 2003	Wistar rats	Morris water maze	Orexin-A	2 h before training	OX-A impairs memory formation
Akbari et al., 2006	Wistar rats	Morris water maze	SB-334867	Before training/ after training/ 15 min before the test	Antagonist impaired memory formation at all stages
García-Brito et al., 2018	Wistar rats	Morris water maze	SB-334867	After training	Antagonist impaired memory consolidation in the 2-day protocol, but not in the 6-day protocol
Ardeshiri et al., 2019	Wistar rats	Morris water maze	SB-334867 TCS-OX2-29	After training for 3 days / 15 min before the test	Antagonists impaired memory consolidation but not retrieval
García-Brito et al., 2020	Wistar rats	Morris water maze	SB-334867	After training	Antagonist impaired memory consolidation, which was ameliorated by ICSS immediately after injections
Han et al., 2020	Wistar rats (SPS- Single Prolonged Stress)	Morris water maze	Orexin-A	Each day of training for 6 days	OX-A ameliorated memory deficits in SPS rats and partially reversed the compensating upregulation of receptor expression

## 4. Conclusion

The lateral hypothalamus/perifornical area, a single source of orexinergic innervation in the brain, exerts its influence on numerous structures engaged in threat and spatial memory processes. In the basolateral amygdala it may influence the modification of sensory inputs from cortical processing areas, changing the amygdalar output, which further influences the hippocampus directly and indirectly via the entorhinal cortex (EC) (Green, 1964). The EC also plays a role in the encoding and consolidation of inhibitory avoidance memories (Izquierdo and Medina, 1997; Izquierdo et al., 2006), although the exact effect of orexins on this structure is still unknown. The hippocampus is a center for the initial formation of traces that are relayed further to cortical areas and then strengthened to form stable engrams, and can be replayed during retrieval. It receives a dense orexinergic input as well as inputs from other structures modified by orexins. More precise experiments further utilizing optogenetic techniques, DREADD, and c-Fos expression may lay the foundations for a deeper understanding of the subtle relationships between certain parts of the hippocampal structure. The role of the noradrenergic locus coeruleus was partly explained. It directly modulates emotional arousal and memory formation in the basolateral amygdala and central amygdalar nucleus (Berridge and Waterhouse, 2003). On the same note, the serotonergic dorsal raphe nucleus modulates the sensory input to the amygdala (Stutzmann et al., 1998), and further research on different memory formation phases could potentially better explain its mechanisms. Different parts of the brain also known to receive orexinergic innervation may participate in memory processes in ways we are yet to unravel. For example, the histaminergic tuberomammillary nucleus (TMN) is thought to facilitate the consolidation of avoidance memory and cued fear conditioning in the basolateral amygdala, CA1, and prefrontal cortex through H1 and H2 receptors (da Silva et al., 2006; Benetti et al., 2012; Fiorenza et al., 2012; Benetti and Izquierdo, 2013), but how orexin receptors in TMN fit into these processes is unknown. Pathological changes in the orexinergic system are associated with overreactions to certain stimuli during PTSD and may partly contribute to memory impairments that occur in patients with Parkinson’s disease, generalized anxiety disorder, major depressive disorder, dementia, and chronic stress. Regulating the biochemistry of orexins with their analogues or antagonists could prevent these symptoms or mitigate existing diseases.

**FIGURE 2:**
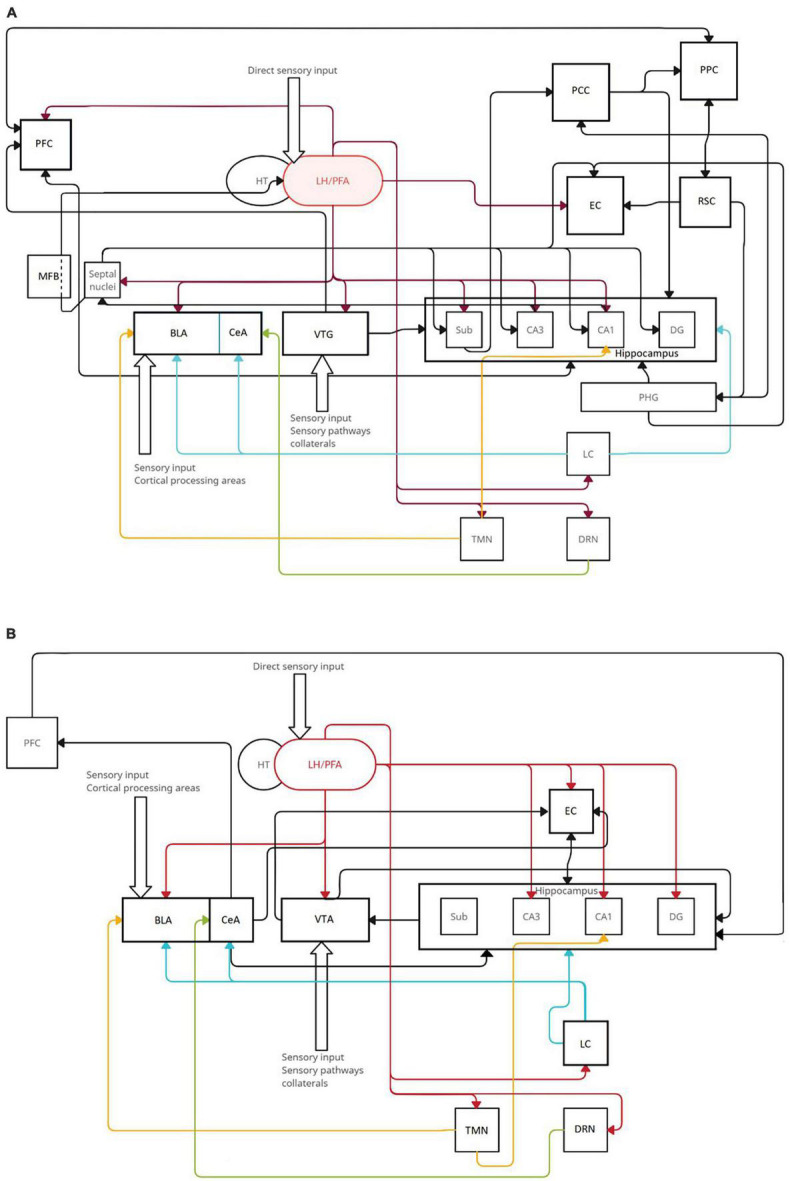
The range of orexinergic fibers in circuitry involved in threats **(A)** and spatial memory **(B)**. Orexins from LH/PFA modulate the actions of various structures, including those that are the primary sources of several neurotransmitters: tuberomammillary nucleus (TMN, yellow histaminergic fibers), locus coeruleus (LC, blue noradrenergic fibers), and dorsal raphae nucleus (DRN, green serotonergic fibers). Black arrows indicate basic neuronal connections included in memory circuits that do not originate from specified neurotransmitter sources. **(A)** The most prominent orexinergic control comes from input to the VTA, BLA, EC, CA1, CA3, and DG. **(B)** Orexins are involved in spatial memory mostly through input to the PFC, subiculum, CA3, CA1, BLA, septal nuclei, and VTA.

When navigating a novel environment, various brain structures participate in processing this environment, creating internal maps and relationships between landmarks, objects, and oneself and finally transforming the experience into spatial memories. The complexity of these processes has been partly elucidated, and the circuitry behind them can be reverse-engineered. The place cells of CA1 and CA3 are thought to be at the center of allocentric processing, creating a cognitive map of the environment and placing oneself within this map, and repeated exposure to the same surroundings stabilizes their neural architecture (Coughlan et al., 2018; Wang et al., 2020). The lateral hypothalamus sends orexinergic fibers to both these hippocampal regions and directly regulates their actions mostly through OX1R. It also innervates the entorhinal cortex, where grid cells create grid-like representations of distinct locations within larger spaces and form possible routes between them (Coughlan et al., 2018). In both fear and spatial memory, orexinergic fibers modify the output of the amygdala as a part of the reinforcement circuit (Sil’kis, 2013). However, the amygdala can act in two ways depending on the state of the brain. In standard conditions, orexins stimulate the influence of the amygdala on the hippocampus, strengthening memory encoding and consolidation (Ardeshiri et al., 2017). On the other hand, in pathological conditions such as PTSD, acute, or chronic stress, the orexinergic activation of the amygdala may impair spatial memory formation, as it reinforces the circuitry involved in stressful events (Morris, 1984; Kim et al., 2001). Orexin A increases the excitation of prefrontal cortex pyramidal neurons, which participate in creating egocentric maps directly and indirectly via dopaminergic fibers originating from the ventral tegmental area. Egocentric processing, which navigates the environment in a self-centered framework depending on the individual viewpoint and body movements, is mainly supported by parietal regions that encode self-representations and self-motion based on the relative position of the starting and ending point of the route (Lyamzin and Benucci, 2019). In the retrosplenial cortex during spatial learning, an experience-dependent memory trace is formed, and the posterior cingulate cortex acts as a hub for inputs from the hippocampus and anterior thalamic nuclei. Both of these structures project to the parahippocampal gyrus, which forms contextual associations (Aminoff et al., 2007). There is no consensus concerning the histamine-releasing neurons of TMN. A few studies have shown that histamine facilitates spatial memory (Chen et al., 2001; Huang et al., 2003), while others have shown that it mediates the effects of novelty (Zlomuzica et al., 2008). The known circuitry of threat and spatial memory is shown in Figure 2. Therefore, research focused on these distinct structures could potentially contribute to a deeper understanding of orexinergic mechanisms in memory processes. Studying those processes *in vivo* was always dependent on the present state of research animal models, and the data received from behavioral tests will, in fact, reflect memory processes in that state. In conclusion, the behavioral experiments concerning the orexinergic system should utilize standardized models and methods with appropriate safeguards regarding the effects of orexins on different physiological functions, taking into account their role in perception, attention, locomotion, sleep regulation, and eating by examining these functions in control group animals. It seems that numerous questions still require answering, but the idea of improved control over the retrieval or extinction of memories, especially associated with threats, is quite appealing. The most important deduction from the literature is that more research is needed using specific animal models for different disorders.

## Author contributions

FM: conception and design of study, acquisition of data, and drafting the manuscript. FM and JC: analysis and/or interpretation of data, revising the manuscript critically for important intellectual content, and approval of the version of the manuscript to be published. Both authors contributed to the article and approved the submitted version.
